# The association of dietary inflammatory potential with skeletal muscle strength, mass, and sarcopenia: a meta-analysis

**DOI:** 10.3389/fnut.2023.1100918

**Published:** 2023-05-15

**Authors:** Haibin Xie, Haochen Wang, Ziying Wu, Wei Li, Yanzhe Liu, Ning Wang

**Affiliations:** ^1^Department of Orthopaedics, Xiangya Hospital, Central South University, Changsha, China; ^2^Hunan Key Laboratory of Joint Degeneration and Injury, Xiangya Hospital, Central South University, Changsha, China; ^3^National Clinical Research Center of Geriatric Disorders, Xiangya Hospital, Central South University, Changsha, China; ^4^Key Laboratory of Aging-Related Bone and Joint Diseases Prevention and Treatment, Ministry of Education, Xiangya Hospital, Central South University, Changsha, China

**Keywords:** dietary inflammatory index (DII), sarcopenia, muscle, meta-analysis, nutrition

## Abstract

**Aims:**

Evidence suggested that dietary inflammatory potential may be associated with age-related skeletal muscle decline, but the results remained controversial. To summarize the evidence for the relationships between dietary inflammatory potential and skeletal muscle strength, mass, and sarcopenia in adults we conducted this meta-analysis.

**Methods:**

Embase, Pubmed, and Web of Science were searched from inception up to 12 March 2023 for studies that evaluated the associations of dietary inflammatory potential [estimated by the Dietary inflammatory index (DII)] with skeletal muscle strength, mass, and sarcopenia. A meta-analysis was then performed to calculate the pooled regression coefficient (β) and odds ratio (OR). The non-linear dose-response relation between DII and sarcopenia was assessed using random-effects dose-response meta-analysis.

**Results:**

This meta-analysis included 24 studies involving 56,536 participants. It was found that high DII was associated with low skeletal muscle strength [OR 1.435, 95% confidence interval (CI) 1.247–1.651, *P* < 0.001, *I*^2^ = 4.97%]. There was a negative association of DII with skeletal muscle strength (β−0.031, 95% CI −0.056 to −0.006, *P* = 0.017, *I*^2^ = 72.69%). High DII was also associated with low skeletal muscle mass (OR 1.106, 95% CI 1.058–1.157, *P* < 0.001, *I*^2^ = 0%). DII had a negative relationship with skeletal muscle mass with high heterogeneity (β−0.099, 95% CI −0.145 to −0.053, *P* < 0.001, *I*^2^ = 88.67%); we downgraded the inconsistency in the subgroup analysis of overweight/obese participants (β−0.042, 95% CI −0.065 to −0.019, *I*^2^ = 12.54%). Finally, the pooled results suggested that high DII was significantly associated with sarcopenia with significant heterogeneity (OR 1.530, 95% CI 1.245–1.880, *P* < 0.001, *I*^2^ = 69.46%); age and BMI may contribute partially to the heterogeneity since heterogeneity was decreased in the subgroup of older age (OR 1.939, 95% CI 1.232–3.051, *I*^2^ = 0%) and the group of overweight/obesity (OR 1.853, 95% CI 1.398–2.456, *I*^2^ = 0%). There was a non-linear dose-response association between DII and sarcopenia (*P* = 0.012 for non-linearity).

**Conclusion:**

This meta-analysis suggested that higher dietary inflammatory potential was significantly associated with lower skeletal muscle strength, mass, and risk of sarcopenia. Future studies with consistent assessment and standardized methodology are needed for further analysis.

## 1. Introduction

Muscle strength plays a critical role in physical function, independence, and vitality in the aged (1, 2), and could predict subsequent health status of the older population and even the risk of mortality (3–7). Loss of muscle strength began at around the 30s and those in their 80s could lose up to 40% of their muscle strength compared with their 20s (8). Reduction of muscle mass, one of the hallmarks of aging, combined with the loss of muscle strength, is referred to as sarcopenia. Sarcopenia as an independent condition recognized by World Health Organization (9), was reported to affect 10% to 27% of people older than 60 years globally and the number of individuals affected by this condition was deemed to increase with the population aging (10). However, a clear understanding of the risk factors causing age-related skeletal muscle loss has not yet been developed, and it remains critical need to identify modifiable risk factors in order to guide the formulation of skeletal muscle loss prevention strategies. In this regard, chronic inflammation has been accepted as one of the accelerating factors causing skeletal muscle loss and sarcopenia (11–15).

Diet patterns have been shown to modulate inflammation and may therefore have different effects on skeletal muscle in view of their inflammatory potential (16–18). The inflammatory potential of diet patterns can be estimated using a validated tool, namely the Dietary inflammatory index (DII) (19). DII was derived from literature review of up to 2,000 research articles. It estimated the association of different food components (45 food components consisting of whole foods, nutrients as well as bioactive compounds) with six serum inflammatory cytokines [i.e., Interleukin (IL)-1β, IL-4, IL-6, IL-10, tumor necrosis factor (TNF)-α, and C-reactive protein (CRP)] (19). In general, a more pro-inflammatory diet corresponds to a higher DII score, while an anti-inflammatory diet corresponds to a lower DII score. However, the results of previous studies on the associations between DII and skeletal muscle strength, mass, and sarcopenia were inconsistent (20–25).

Meta-analysis as an effective means to synthesize the existing evidence may help fill this knowledge gap. Recently, a meta-analysis including 11 studies suggested that DII may be associated with sarcopenia (26). However, it did not conduct subgroup analyses based on diet and muscle mass assessment methods, which were two of the important sources of inter-study heterogeneity and bias for the association between DII and skeletal muscle. Besides, it only investigated sarcopenia, leaving the effect of DII on muscle strength and mass unclear. Therefore, a meta-analysis with more comprehensive included studies is necessary to further elucidate the association of DII with skeletal muscle strength, mass, and sarcopenia.

## 2. Methods

### 2.1. Protocol and registration

This study was reported according to the Preferred Reporting Items for Systematic Review and Meta-Analysis (PRISMA) (Supplementary Appendix 1) (27) and Meta-analyses Of Observational Studies in Epidemiology (MOOSE) guideline (Supplementary Appendix 2) (28). The protocol was prospectively registered in PROSPERO (CRD42022334333).

### 2.2. Search strategy

Three databases, namely Embase, Pubmed, and Web of Science, were searched from inception to March 12, 2023. The search strategy was constructed based on following keywords: (“DII” OR “dietary inflammatory index” OR “inflamma* AND diet”) AND (“sarcopen*” OR “sarcopenia” OR “sarcopenic” OR “muscle mass” OR “muscle volume” OR “muscle quality” OR “muscle size” OR “lean mass” OR “grip strength” OR “hand strength” OR “muscle strength” OR “gripping strength” OR “holding power” OR “grip dynamometer” OR “handgrip” OR “muscular atrophy” OR “muscular dystrophy” OR “muscle dystrophy” OR “muscle atrophy”). A systematic search strategy was designed as broad as possible and adjusted according to databases (Supplementary Appendix 3). No language restriction was applied, and Google Translate was used for non-English articles (29). Reference manager software was applied to automatically remove duplicates. For finally included studies, the reference lists and related reviews were manually screened for additional studies meeting the eligibility criteria.

### 2.3. Eligibility criteria

The research question was specified using PICO (Supplementary Appendix 4). The inclusion criteria for studies were: (1) participants were adults aged 18 years or older; (2) intervention or exposure was diet patterns with different dietary inflammatory potential (evaluated by DII or energy-adjusted DII [E-DII]); (3) groups with high DII were compared with those with low DII; (4) outcomes included skeletal muscle strength, mass, and sarcopenia; (5) studies with observational study design (e.g., cross-sectional studies, case-control studies, and longitudinal studies).

### 2.4. Study selection and data extraction

After removal of duplicates, two investigators screened the titles and abstracts and then conducted full-texted assessment on the included studies independently. The agreement between two authors was acceptable for the titles and abstracts screening (Kappa statistic was 0.85), when disagreement was solved by discussion, and complete agreement was achieved in full-text assessment (Kappa statistic was 1.0). The desired data was extracted using a standardized table, which included study characteristics (i.e., author, year of publication, country, study type, and study setting), demographic information of participants [i.e., age, sex, and body mass index (BMI)], exposure measurements (DII reported as continuous variables, or category variables, for which the methods used for categorization were extracted as well), outcome measurements (muscle components reported as continuous variables, or category variables, and for which the methods used for categorization were extracted as well), effect sizes, and adjustments.

We extracted the adjusted odds ratio (OR) when comparing different DII category groups with the lowest group, or a β coefficient for the continuous association between DII and skeletal muscle. If a study reported results from diverse models with adjustments of different potential confounding factors, the most adjusted results would be chosen (30). We contacted the authors of studies with missing data for further information.

### 2.5. Quality assessment and certainty of evidence

The methodological quality was assessed by the Risk Of Bias In Non-randomized Studies (ROBINS-E) assessment tool. ROBINS-E was based on 7 domains including risk of confounding bias, selection bias, exposure measurement, departure from intended exposure bias, missing data, outcome measurement, and selection of reported bias (31). Articles were judged as low risk of bias if all criteria were low risk of bias, if at least one criterion was rated as moderate, serious, or critical risk of bias, the overall quality of study would be regarded as moderate, high, and very high risk of bias, respectively.

Two investigators assessed the methodological quality of each study independently. Any disagreement was resolved by discussion as far as possible; if failed, the corresponding author (Ning Wang) would be consulted to help make the final decision.

Certainty of evidence was assessed by using the Grades of Recommendation, Assessment, Development and Evaluation (GRADE) tool. Factors including within-study risk of bias, the indirectness of the evidence, heterogeneity, precision of the effect or association estimates, and publication bias were considered to reach an overall certainty of the evidence rating of very low, low, moderate, or high for each outcome (32).

### 2.6. Statistical analysis

We grouped studies according to the methods used for reporting DII exposure and skeletal muscle outcomes. Three main methods of reporting results were used: (1) β coefficient for the continuous association between DII exposure and skeletal muscle strength or mass; (2) ORs for the risk of low muscle strength or mass comparing participants having the highest DII with those having the lowest DII; and (3) ORs for the risk of sarcopenia comparing participants having the highest DII with those having the lowest DII.

Random-effects model assumes that different studies estimate different but related effects, and yields identical results as the fixed-effects model in the absence of heterogeneity. Therefore, to obtain conclusions generalized to wilder arrays of situations, we used random-effects model in all analyses (33–35). The statistical heterogeneity of the included studies was examined by Cochrane’s Q test and *I*^2^ (*P* < 0.05 indicated statistically significant heterogeneity and *I*^2^ > 50% indicated high-degree heterogeneity (36).

For studies reporting relative risks of sarcopenia for several categories with number of cases and controls, we further conducted a dose-response analysis (37). The median or mean of DII for each category was assigned to each corresponding OR. The dose-response meta-analysis was conducted using restricted cubic spline models with 3 knots to estimate potential non-linear trend in each study, and the results from included studies were pooled using random-effect multivariate meta-analysis (38).

Since the predictive ability of DII scores based on 27–45 food parameters were validated in previous studies (22, 39), sensitivity analyses omitting studies that calculated DII scores based on less than 27 components were performed. Additional sensitivity analyses were performed by removing each single study from the analysis to assess the robustness of the findings. Subgroup studies were carried out according to geographic region (different continents), age (<65 and ≥65 years old), BMI [overweight/obese (BMI ≥ 25kg/m^2^) and normal/underweight (BMI < 25kg/m^2^)], diet assessment methods, muscle mass assessment methods, and the definition of sarcopenia [European Working Group on Sarcopenia in Older People (EWGSOP), Asian Working Group for Sarcopenia (AWGS), Foundation for The National Institute of Health (FNIH), and low appendicular skeletal muscle mass (ASM)]. Since several studies did not present the mean BMI of participants, they were precluded in the subgroup analyses for BMI. The risk of publication bias was analyzed by funnel plots with Egger test, using Duval and Tweedie trim-and-fill method for adjustment of funnel plot asymmetry (40, 41). *P* values less than 0.05 were considered statistically significant. Comprehensive Meta-Analysis (CMA) software V.3.3.0 (Biostat) was used for meta-analysis and Stata V.14 was used for dose-response meta-analysis and publication bias assessment.

## 3. Results

### 3.1. Study selection and characteristics

**FIGURE 1:**
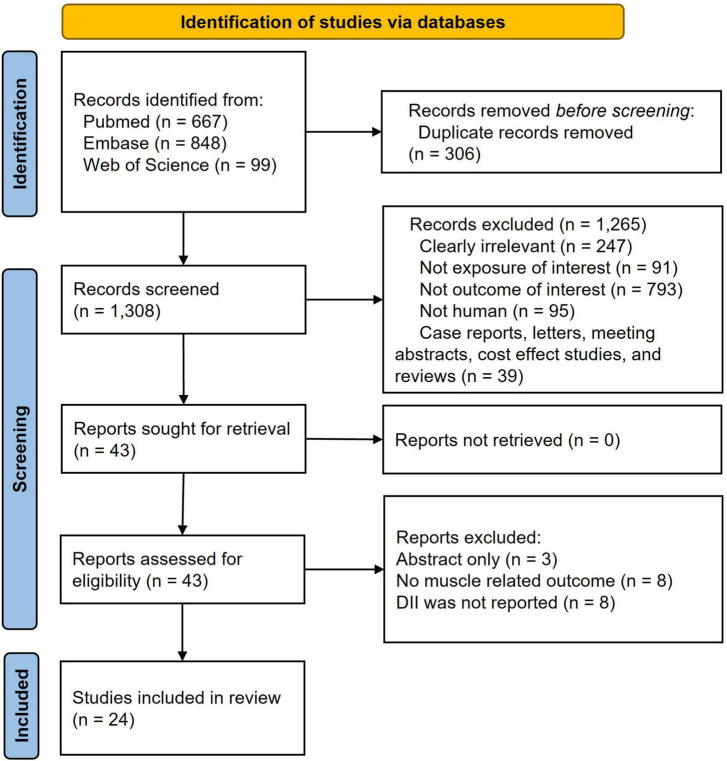
Preferred reporting items of systematic reviews and meta-analysis (PRISMA) flow chart.

Of the 1,308 records identified by initial search after removal of duplicates, 1,265 obviously ineligible studies were excluded in the titles and abstracts screen, resulting in 43 articles for full-text assessment. Eventually, 24 researches involving 56,536 participants met the eligibility criteria (Figure 1). Twenty studies were cross-sectional designed and four were longitudinal studies (20–25, 42–59). No interventional study was available for this meta-analysis.

Table 1 and Supplementary Appendix 5 summarized the detailed characteristics of the included studies. The number of participants ranged from 79 to 25,781, with the mean age ranging from 32.6 to 81.1 years. The mean BMI was higher than 25 kg/m^2^ in 13 studies (54.2%) and lower than 25 kg/m^2^ in 7 studies (29.2%), and the remaining 4 studies (16.7%) did not report a mean BMI. The largest proportion of studies (41.7%) were carried out in Asia, followed by North America (29.2%). The choice of measurement of skeletal muscle mass was also different, with DXA (80%) being the most widely used, followed by bio-impedance analysis (BIA) (20%). The handgrip strength was the most frequent choice of measurement of skeletal muscle strength. Low muscle strength and mass were defined either by recommended cut-off values for the participants or based on the population-specific thresholds. The diagnosis of sarcopenia varied across five different diagnostic criteria, including EWGSOP 1, EWSOP 2, FNIH, AWGS 2019, and a criterion based on low ASM alone. Among them, AWGS 2019 (35.7%) was mostly used. The ROBINS-E tool suggested moderate to high risk of bias for most studies, and very high risk of bias for one study. In most included studies, bias originated from uncontrolled confounding bias and missing data (Supplementary Appendix 6).

**TABLE 1:** Characteristic of included studies.

Study	Country	Study type	Participants’ health	Female (%)	Mean age (year)	BMI (kg/m^2^)	Diet assessment	Comparison level	Outcome	Adjusted confounders
Bagheri et al. (24)	Iran	Cross-sectional	Without conditions causing sarcopenia other than age	50	66.7	27.4	FFQ	Top vs. bottom tertile	Low muscle strength/mass, sarcopenia (category)	Age, sex, energy intake, physical activity, smoking, alcohol consumption, medication use, and history of disease
Bian et al. (20)	China	Cross-sectional	Crohn’s disease	27.86	32.6	20.7	FFQ	Top vs. bottom quartile	Low muscle strength/mass, sarcopenia (category)	Age, sex, BMI, smoking, alcohol consumption, nutritional status, Crohn’s disease activity, and energy intake
Cervo et al. (43)	Canada	Cross-sectional	Community-dwelling older men without critical ill	0	81.1	27.7	Diet history questionnaire	Continuous	Muscle mass (continuous)	Age, smoking, calcium intake, physical activity, use of NSAIDs, use of bisphosphonates, presence of musculoskeletal disease, and comorbidity
Cervo et al. (42)	Australia	Cross-sectional	Community-dwelling older adults	51.14	63.0	27.9	FFQ	Continuous	Muscle strength/mass (continuous)	Age, percent body fat, smoking, steps per day, calcium, and alcohol intakes
Chen et al. (44)	USA	Cross-sectional	Community-dwelling adults	48.01	37.2	NA	24 h-dietary recall	Continuous/top vs. bottom tertile	Muscle strength/mass (continuous), and sarcopenia (category)	Age, sex, race, educational level, marriage, family poverty income ratio, smoking, drinking, physical activity, BMI, diabetes, and hypertension
Chen et al. (45)*	USA	Cross-sectional	Community-dwelling adults	51.0	62.1	27.7	24 h-dietary recall	Continuous/top vs. bottom tertile	Muscle strength/mass (continuous), low muscle mass (category), and sarcopenia (category)	Age, sex, race, education, marital status, nativity, smoking, physical activity, BMI, chronic disease, and protein
Davis et al. (46)	Australia	Longitudinal	Community-dwelling women	100	50.3	26.0	DQES	Continuous	Muscle mass (continuous)	Age, physical activity, smoking, protein, dietary energy
Davis et al. (47)*	Australia	Longitudinal	Community-dwelling adults	0	50.0	26.5	FFQ	Continuous	Muscle mass (continuous)	Age, fat mass, and physical activity
Esmaeily et al. (48)	Iran	Cross-sectional	Community-dwelling adults	66.0	77.0	29.0	FFQ	Continuous/top vs. bottom tertile	Muscle strength (continuous) and low muscle strength (category)	Age, family number, gender, CVD medication, BMI, and physical activity
Geng et al. (21)	USA	Cross-sectional	Community-dwelling adults	45.23	45.4	NA	24 h-dietary recall	Top vs. bottom tertile	Sarcopenia (category)	Age, gender, race, ratio of family income to poverty, education level, marital, BMI, comorbidity, smoking, alcohol, physical activity
Gojanovic et al. (49)	Australia	Cross-sectional	Community-dwelling adults	34.36	66.4	27.7	DQES	Continuous	Muscle mass (continuous)	Age, sex, and body fat percentage
Haß et al. (50)	German	Cross-sectional	Healthy old adults	75	72.4	28.8	24 h-dietary recall	Continuous	Muscle strength/mass (continuous)	Age, sex, and physical activity
Huang et al. (22)	USA	Cross-sectional	Chronic kidney disease patients	54.89	55.6	NA	24 h-dietary recall	Continuous/top vs. bottom tertile	Muscle mass (continuous) and sarcopenia (category)	Age, gender, race, income, physical activity, smoking, alcohol, diabetes, hypertension, overweight, central obesity, comorbidity, eGFR, ACR, hypoalbuminemia, low energy intake, low protein intake, CRP, WBC, NLR, and NHANES strata
Inoue et al. (51)	Japan	Cross-sectional	Ambulatory patients aged 65 years or older without obvious disability due to certain disease	77.6	67.4	24.1	BDHQ	Top vs. bottom quartile	sarcopenia, low muscle strength and mass (category)	Age, sex, comorbidity, physical activity, BMI, protein intake, and energy intake
Jin et al. (52)	Korea	Cross-sectional	Community-dwelling menopause women	100	63.5	NA	3-days food record	Continuous	Muscle strength/mass (continuous)	Age, BMI, menopausal period, smoking, alcohol, vitamin D supplement intake, and physical activity.
Kim and Park (53)	Korea	Cross-sectional	Community-dwelling adults	61.1	76.9	24.6	24 h-dietary recall	Not reported	Low muscle strength (category)	Age, chewing ability, and energy intake
Laclaustra et al. (54)	Spain	Longitudinal	Community-dwelling adults	51.5	68.4	28.5	Computer-based diet history	Top vs. bottom tertile	Low muscle strength (category)	Age, sex, education, smoking, BMI, energy intake, comorbidity, time spent watching TV, and physical activity
Linton et al. (58)	Australia	Cross-sectional	Functionally able, community-dwelling adults	76.4	72.1	25.8	24 h-dietary recall	Continuous	Muscle strength/mass (continuous)	Age, gender, waist circumference, comorbidity, and physical activity
Park et al. (23)	USA	Cross-sectional	Community-dwelling women	100	62.3	25.7	24 h-dietary recall	Top vs. bottom halves	Sarcopenia (category)	Age, family income, regular exercise, education, smoking and female hormone supplements
Son et al. (25)	Japan	Cross-sectional	Community-dwelling adults	48.2	74.6	22.2	BDHQ	Top vs. bottom tertile	Low muscle strength/mass (category) and sarcopenia (category)	Age, education, protein intake, physical activity, comorbidity, eating alone, Lubben Social Network Scale (LSNS) social ties (< 12), Geriatric Depression Scale (GDS) ≥ 6, and Geriatric Oral Health Assessment Index (GOHAI) score
Song et al. (55)	Korea	Cross-sectional	Community-dwelling women	100	57.7	24.3	3-days food record	Top vs. bottom tertile	Muscle mass (continuous)	Age, menopausal period, smoking, alcohol, BMI, and physical activity
Su et al. (56)	China	Longitudinal	Community-dwelling adults	50	72.5	23.7	FFQ	Top vs. bottom tertile	Sarcopenia (category)	Age, BMI, smoking, comorbidity, vitamin D status, and physical activity
Su et al. (57)*	China	Cross-sectional	Community-dwelling adults	52.5	71.9	23.7	FFQ	Continuous	Muscle strength/mass (continuous)	Age, corresponding measurement, BMI, smoking, physical activity, previous fracture, hypertension, diabetes, chronic obstructive lung disease, cardiovascular disease, rheumatoid arthritis, non-steroidal anti-inflammatory agent use, and osteoporosis medication
Xie et al. (59)	USA	Cross-sectional	Community-dwelling adults	51.0	51.7	29.2	24 h-dietary recall	Continuous	Muscle strength (continuous)	Age, gender, race, education, marital status, physical activity, energy intake, smoking

FFQ, Food Frequency Questionnaires; DQES, Dietary Questionnaire for Epidemiological Studies; BDHQ, brief self-administered diet history questionnaire; DII, Dietary inflammatory index; E-DII, energy-adjusted DII; SMI, skeletal muscle index; TUG, Up-ana-Go test; NA, not available. Symbol * was used to identify different articles with the same author surnames and publication time.

### 3.2. Association between DII and skeletal muscle strength

**FIGURE 2:**
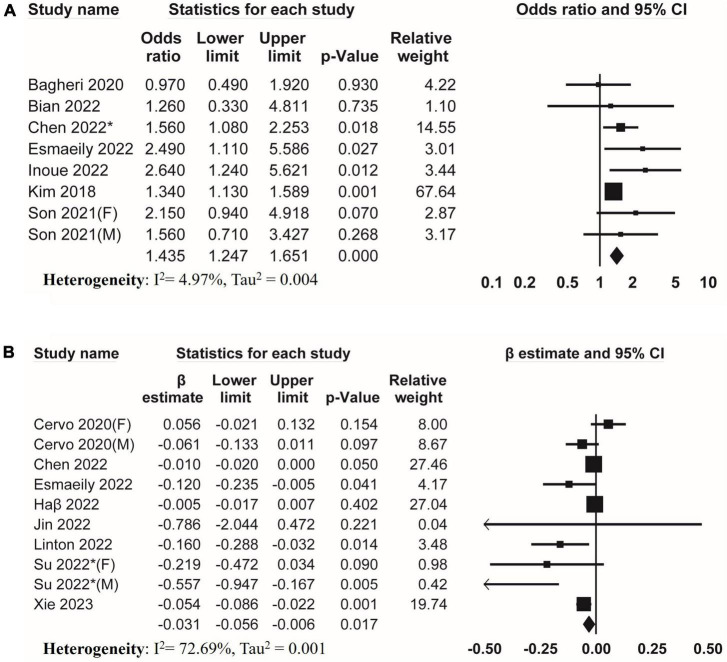
Forest plots of meta-analyses on the association between DII and muscle strength. **(A)** Forest plot of meta-analysis on the association between DII and risk of low muscle strength; **(B)** forest plot of meta-analysis on the association between DII and muscle strength.

Seven studies investigated the association between DII and low skeletal muscle strength. The result of meta-analysis revealed a positive association between DII and low skeletal muscle strength (pooled OR = 1.435, 95% CI, 1.247–1.651) (Figure 2A) without evidence of substantial heterogeneity (*I*^2^ = 4.97%, Tau^2^ = 0.004). Eight studies reported β from multiple linear regression. The result of meta-analysis of these studies showed a negative association of DII with skeletal muscle strength (pooled β = −0.031, 95% CI, −0.056 to −0.006, *I*^2^ = 72.69%, Tau^2^ = 0.001) (Figure 2B).

### 3.3. Association between DII and skeletal muscle mass

**FIGURE 3:**
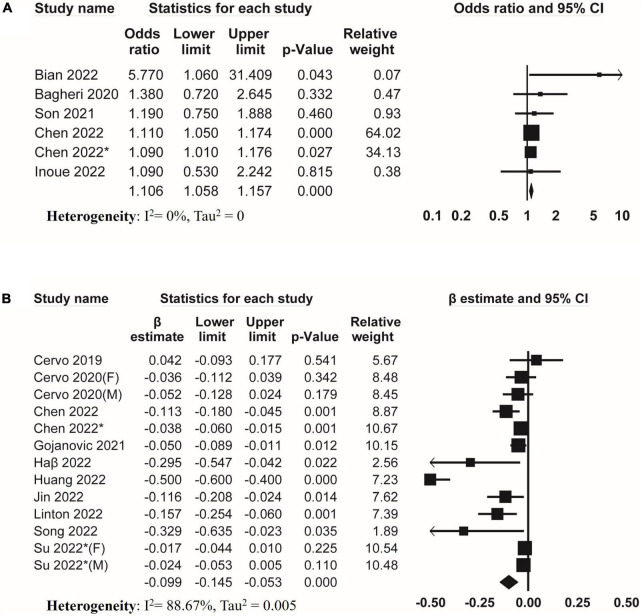
Forest plots of meta-analyses on the association between DII and muscle mass. **(A)** Forest plot of meta-analysis on the association between DII and risk of low muscle mass; **(B)** forest plot of meta-analysis on the association between DII and muscle mass.

Regarding skeletal muscle mass, six studies investigated the association between DII and low skeletal muscle mass (Figure 3A). The result of meta-analysis suggested that DII was associated with low skeletal muscle mass (pooled OR = 1.106, 95% CI, 1.058–1.157) without evidence of heterogeneity (*I*^2^ = 0%, Tau^2^ = 0). Moreover, meta-analysis of eleven studies suggested that DII was negatively associated with muscle mass (pooled β = −0.099, 95% CI, −0.145 to −0.053) with significant heterogeneity (*I*^2^ = 88.67%, Tau^2^ = 0.005) (Figure 3B).

### 3.4. Association between DII and sarcopenia

**FIGURE 4:**
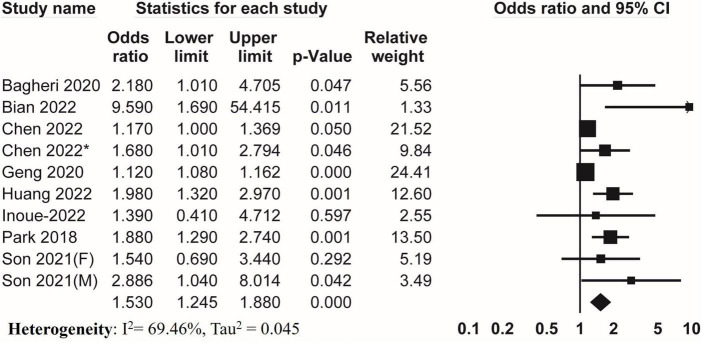
Forest plots of meta-analyses on the association between DII and sarcopenia.

Nine studies examined the association between DII and sarcopenia. Meta-analysis of these studies covering 41,233 participants revealed that higher DII was associated with an increased risk of sarcopenia (pooled OR = 1.530, 95% CI, 1.245–1.880) (Figure 4). An *I*^2^ of 69.46% with Tau^2^ of 0.045 indicated significant heterogeneity among studies.

**FIGURE 5:**
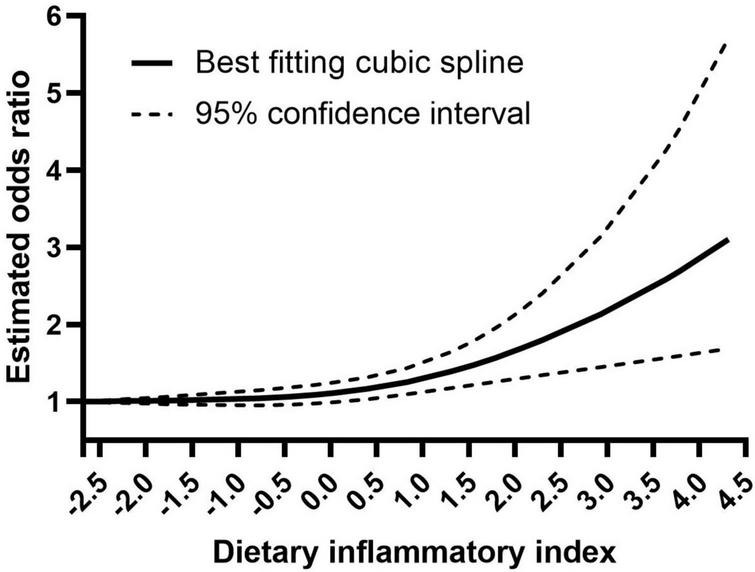
Dose-response analysis of the association between DII and sarcopenia.

For dose-response meta-analysis, we included seven studies that divided DII into at least three categories, and two studies were excluded (23, 44). Setting the reference DII level as −2.68, we found a significant non-linear dose-response relationship between DII and sarcopenia (*P* = 0.012 for non-linearity). No increased risk was observed with higher DII when DII was lower than 0. The risk of sarcopenia increased significantly with DII when DII was at the range of 1–4.315 (Figure 5).

### 3.5. Sensitivity analyses

Sensitivity analyses omitting studies that calculated DII scores based on less than 27 food parameters altered the results on low muscle mass without significant impact on the heterogeneity (Table 2). In addition, omitting each single study did not alter the findings (Supplementary Appendix 7), suggesting that the results were robust.

**TABLE 2:** Sensitivity analyses omitting studies using less than 27 components in DII calculation.

Outcome	Number	Pooled OR (95% CI)	Pooled β (95% CI)	*P*-value for estimated effect	*I*^2^ (%)	Tau^2^
Low muscle strength	3	1.314 (1.115, 1.548)	–	**0.001**	0.00	0.00
Muscle strength	5	–	−0.074 (−0.136, −0.012)	**0.019**	78.22	0.00
Low muscle mass	3	1.339 (0.819, 2.186)	–	0.244	50.64	0.10
Muscle mass	6	–	−0.156 (−0.247, −0.066)	**0.001**	93.97	0.01
Sarcopenia	6	1.475 (1.169, 1.861)	–	**0.001**	78.59	0.05

OR, odds ratio. Values in bold indicated statistically significant results.

### 3.6. Subgroup analyses

We further conducted subgroup analyses by stratifying studies according to geographic regions, age, BMI, diet assessment methods, muscle mass assessment methods, and sarcopenia diagnostic criteria (Table 3), and the results were reported according to outcomes as follows:

**TABLE 3:** Subgroup analyses.

			Pooled results of subgroups		Heterogeneity of subgroups	
**Outcome and subgroup**	**Classification**	**No**	**OR/β (95% CI)**	***P*-value**	***I*^2^(%)**	**Tau^2^**
**Low muscle strength**						
Continents	Asia	6	1.507 (1.191,1.907)	**0.001**	15.88	0.02
	North America	1	1.560 (1.080, 2.253)	**0.018**	–	–
Age	≥65 years	5	1.565(1.182, 2.071)	**0.002**	29.61	0.04
	<65 years	2	1.537 (1.078, 2.191)	**0.018**	0	0.00
BMI	Overweight/Obesity	3	1.515 (0.994, 2.310)	0.053	35.80	0.05
	Normal/underweight	4	1.428 (1.198, 1.702)	**<0.001**	1.89	0.00
Diet assessment method	24 h-dietary recall	3	1.403 (1.208, 1.629)	**<0.001**	0.00	0.00
	FFQ	3	1.443 (0.765, 2.720)	0.257	35.14	0.11
	BDHQ	1	2.640 (1.240, 5.621)	**0.012**	–	–
**Muscle strength**						
Continents	Asia	3	−0.246 (−0.446, −0.045)	**0.016**	46.13	0.02
	Australia	2	−0.046 (−0.158, 0.065)	0.415	78.92	0.01
	Europe	1	−0.005 (−0.017, 0.007)	0.402	–	–
	North America	2	−0.029 (−0.072, 0.014)	0.180	84.89	0.00
Age	≥65 years	3	−1.41 (−0.291, 0.010)	0.067	78.80	0.02
	<65 years	5	−0.023 (−0.060, 0.015)	0.243	68.94	0.00
BMI	Overweight/Obesity	6	−0.019 (−0.040, 0.001)	0.058	65.99	0.00
	Normal/underweight	2	−0.244 (−0.426, −0.062)	**0.009**	44.65	0.01
Diet assessment method	24 h-dietary recall	3	−0.016 (−0.033, 0.001)	0.068	74.86	0.00
	FFQ	4	−0.107 (−0.209, −0.005)	**0.039**	74.82	0.01
	3-days food record	1	−0.786 (−2.044, 0.472)	0.221	–	–
**Low muscle mass**						
Continents	Asia	4	1.301 (0.909, 1.864)	0.151	11.00	0.02
	North America	2	1.103 (1.055, 1.154)	**<0.001**	0.00	0.00
Age	≥ 65 years	3	1.214 (0.870, 1.695)	0.254	0.00	0.00
	<65 years	3	1.105 (1.022, 1.195)	**<0.013**	47.42	0.00
BMI	Overweight/Obesity	2	1.094 (1.014, 1.180)	**0.021**	0.00	0.00
	Normal/underweight	3	1.353 (0.761, 2.408)	0.303	39.61	0.11
Diet assessment method	24 h-dietary recall	3	1.104 (1.056, 1.154)	**<0.001**	0.00	0.00
	FFQ	2	2.258 (0.896, 8.559)	0.231	58.10	0.60
	BDHQ	1	1.090 (0.530 2.242)	0.815	−	−
Muscle mass assessment method	DXA	4	1.104 (1.056, 1.155)	**<0.001**	0.00	0.00
	BIA	2	2.105 (0.476, 9.305)	0.326	67.79	0.85
**Muscle mass**						
Continents	Asia	3	−0.040 (−0.082, 0.002)	0.061	61.88	0.00
	Australia	3	−0.063 (−0.104, −0.022)	**0.003**	33.49	0.00
	Europe	1	−0.295 (−0.547, −0.042)	**0.022**	–	–
	North America	4	−0.152 (−0.331, 0.027)	0.095	96.36	0.03
Age	≥65 years	5	−0.043 (−0.080, −0.006)	**0.021**	64.14	0.00
	<65 years	6	−0.150 (−0.251, −0.049)	**<0.004**	92.93	0.02
BMI	Overweight/Obesity	5	−0.042 (−0.065, −0.019)	**<0.001**	12.54	0.00
	Normal/underweight	3	−0.027 (−0.047, −0.008)	**0.007**	73.25	0.00
Diet assessment method	24 h-dietary recall	4	−0.227 (−0.421, −0.033)	**0.022**	96.40	0.04
	FFQ	3	−0.038 (−0.069, −0.007)	**0.017**	49.58	0.00
	3-days food record	1	−0.116 (−0.208, −0.024)	**0.014**	–	–
	BDHQ	1	−0.329 (−0.635, −0.023)	**0.035**	–	–
	Diet history questionnaire	1	−0.042 (−0.093, 0.177)	0.541	–	–
Muscle mass assessment method	DXA	9	−0.089 (−0.135, −0.042)	**<0.001**	89.89	0.01
	BIA	2	−0.309 (−0.503, −0.114)	**0.002**	0.00	0.00
**Sarcopenia**						
Continents	Asia	4	2.164 (1.364, 3.431)	**0.001**	7.46	0.02
	North America	5	1.386 (1.135, 1.692)	**0.001**	76.56	0.03
Age	≥65 years	3	1.939 (1.232, 3.051)	**0.004**	0.00	0.00
	<65 years	6	1.452 (1.164, 1.812)	**0.001**	78.14	0.04
BMI	Overweight/Obesity	3	1.853 (1.398, 2.456)	**<0.001**	0.00	0.00
	Normal/underweight	3	2.260 (1.158, 4.411)	**0.017**	30.56	0.14
Diagnostic criteria	EWGSOP	1	2.180 (1.010, 4.705)	**0.047**	–	–
	Low muscle mass	1	1.880 (1.290, 2.740)	**0.001**	–	–
	AWGS 2019	3	2.260 (1.158, 4.411)	**0.017**	30.56	0.39
	FNIH	4	1.279 (1.067, 1.532)	**0.008**	70.30	0.02
Diet assessment method	24 h-dietary recall	6	1.434 (1.178, 1.746)	**<0.001**	71.19	0.03
	FFQ	2	3.696 (0.920, 14.847)	0.065	57.23	0.63
	BDHQ	1	1.390 (0.410, 4.712)	0.597	−	−
Muscle mass assessment method	DXA	7	1.421 (1.171, 1.725)	**<0.001**	69.90	0.03
	BIA	2	2.730 (1.137, 6.553)	**0.025**	46.50	0.28

No, numbers of studies; OR, odds ratio. Values in bold indicated statistically significant results.

*Low muscle strength*: Geographic regions and age had no impact on the association between DII and low muscle strength, and no significant heterogeneity was observed among these subgroups.

*Muscle strength*: In subgroup analyses, subgroups for Asia (pooled β = −0.246, 95% CI −0.446 to −0.045, studies = 3, *I*^2^ = 46.13%, Tau^2^ = 0.02), normal/underweight (pooled β = −0.244, 95% CI −0.426 to −0.062, studies = 2, *I*^2^ = 44.65%, Tau^2^ = 0.01), and Food Frequency Questionnaires (FFQ) (pooled β = −0.1.07, 95% CI −0.209 to –0.005, studies = 4, *I*^2^ = 74.82%, Tau^2^ = 0.01) revealed a significant association between DII and muscle strength without substantial heterogeneity. We failed to downgrade the heterogeneity in subgroup analyses.

*Low muscle mass*: The significant association between DII and low skeletal muscle mass was altered in subgroup analyses; we found a significant association between DII and low skeletal muscle mass in subgroups for North America (pooled OR = 1.103, 95% CI 1.055–1.154, studies = 2, *I*^2^ = 0%, Tau^2^ = 0), participants younger than 65 years old (pooled OR = 1.105, 95% CI 1.022–1.195, studies = 3, *I*^2^ = 47.42%, Tau^2^ = 0), overweight/obese (pooled OR = 1.094, 95% CI 1.014–1.180, studies = 2, *I*^2^ = 0%, Tau^2^ = 0), 24h-dietary recall (pooled OR = 1.104, 95% CI 1.056–1.154, studies = 3, *I*^2^ = 0%, Tau^2^ = 0), and DXA (pooled OR = 1.104, 95% CI 1.056–1.155, studies = 4, *I*^2^ = 0%, Tau^2^ = 0) without substantial heterogeneity.

*Muscle mass*: In the primary analysis, we observed substantial inconsistency between included studies, but we failed to downgrade the heterogeneity in all subgroups except for the subgroup only including the overweight/obesity population (pooled β = −0.042, 95% CI −0.065 to −0.019 studies = 5, *I*^2^ = 12.54%, Tau^2^ = 0) and subgroup using BIA for muscle mass assessment (pooled β = −0.309, 95% CI −0.503 to −0.114 studies = 2, *I*^2^ = 0%, Tau^2^ = 0).

*Sarcopenia*: Most subgroup analyses did not alter the significant association between DII and sarcopenia except for the subgroup for the groups that used FFQ to assess diet. Only in the subgroup involving older participants (≥65 years) and subgroup of overweight/obese, heterogeneity was reduced, suggesting that age might account for the heterogeneity to a certain extent.

### 3.7. Publication bias

The funnel plots showed asymmetry among outcomes of muscle strength, mass, and sarcopenia (Supplementary Appendix 8) with results of Egger test suggesting evidence of publication bias (Table 4). The adjusted effect estimates showed similar results with primary analyses in outcomes of low muscle strength, low muscle mass while the trim-and-fill analysis alter the significant association of DII with muscle strength and sarcopenia; no adjustment was needed for the analysis of muscle mass (Supplementary Appendix 8 and Table 4).

**TABLE 4:** Risk of publication bias of included studies in meta-analysis based on Egger test and results of trim-and-filled analysis.

	Egger test		Trim-and-fill analysis	
**Outcome**	***t*-Value**	***P*-value**	**Studies trimmed**	**Adjusted estimates**
Low muscle strength	1.46	0.194	3	**1.360 (1.096, 1.687)**
Muscle strength	3.05	**0.016**	4	−0.022 (−0.051, 0.006)
Low muscle mass	1.86	0.136	2	**1.103 (1.029, 1.183)**
Muscle mass	2.35	**0.039**	0	**−0.099 (−0.145, −0.053)**
Sarcopenia	5.38	**0.001**	6	1.149 (0.943, 1.400)

Values in bold indicated statistically significant results.

However, these results should be interpreted with caution since Egger test is not accurate when the number of included studies is small (40, 41).

### 3.8. Certainty of evidence

We assessed the certainty of evidence using GRADE. The association between DII and low muscle mass was of low certainty, and the associations of DII with low muscle strength, muscle strength, muscle mass and sarcopenia were of very low certainty (Table 5).

**TABLE 5:** Summary of findings (Sof) by GRADE system.

Outcome	Studies number	Risk of bias	Inconsistency	Indirectness	Imprecision	Publication bias	Other consideration	Effect size (95% CI)	Certainty^a^
Low muscle strength	7	Serious	Not serious	Not serious	Not serious	Not serious	None	OR: 1.435 (1.247, 1.651)	⊕◯◯◯ Very low due to risk of bias
Muscle strength	8	Serious	Serious	Not serious	Not serious	Serious	None	β: −0.031 (−0.056, −0.006)	⊕◯◯◯ Very low due to risk of bias, inconsistency, and publication bias
Low muscle mass	6	Not serious	Not serious	Not serious	Not serious	Not serious	None	OR: 1.106 (1.058, 1.157)	⊕⊕◯◯ Low
Muscle mass	11	Serious	Serious	Not serious	Not serious	Serious	None	β: −0.099 (−0.145, −0.053)	⊕◯◯◯ Very low due to risk of bias, inconsistency, and publication bias
Sarcopenia	9	Serious	Serious	Not serious	Not serious	Serious	Upgraded for dose-response relationship	OR: 1.530 (1.245, 1.880)	⊕◯◯◯ Very low due to risk of bias, inconsistency, and publication bias

OR, odds ratio.

^a^High certainty: We are very confident that the true effect lies close to that of the estimate of the effect; Moderate certainty: We are moderately confident in the effect estimate. The true effect is likely to be close to the estimate of the effect, but there is a possibility that it is substantially different; Low certainty: Our confidence in the effect estimate is limited. The true effect may be substantially different from the estimate of the effect; Very low quality: We have very little confidence in the effect estimate: The true effect is likely to be substantially different from the estimate of effect.

## 4. Discussion

This meta-analysis explored the associations of DII with skeletal muscle strength, mass, and sarcopenia, and the results showed that DII was correlated with both low skeletal muscle strength and mass. Consistently, a higher DII was associated with an increased risk of sarcopenia. Our dose-response meta-analysis showed that the risk of sarcopenia was at the lowest point when DII was −2.68 to 0, and increased DII raised the risk of sarcopenia when DII was higher than 0.

### 4.1. Comparison with previous studies

An earlier systematic review and meta-analysis reported the association between adherence to a Mediterranean diet and physical performance in older adults (18), while another systematic review by Bloom et al. suggested that a healthier diet was associated with a decreased risk of sarcopenia in the aged people (17). Both studies indicated that diet habits might influence the skeletal muscle condition in older adults. The dietary inflammatory potential has been demonstrated to influence health outcomes as one of the modulators for systematic inflammation (19, 60, 61). Recently, a meta-analysis including 11 studies suggested that DII may be associated with sarcopenia (26). Yet, it did not conduct subgroup analyses based on diet and muscle mass assessment methods, which were two of the important sources of inter-study heterogeneity and bias for the association between DII and skeletal muscle. For example, 24-h recall was less biased than FFQ while FFQ worked better on episodically consumed nutrient and food (62). Moreover, it only investigated sarcopenia, leaving the effect of DII on muscle strength and mass unclear. Given that muscle strength and mass decline at different speeds and independently predispose old adults to risk of adverse events (63), assessing the impact of DII on muscle strength and mass separately is favorable. In response to this situation, our research summarized all the available studies, took these potential confounders into consideration, and provided more comprehensive evidence for the effect of DII on skeletal muscle.

### 4.2. Possible explanations

The DII was formulated based on extensive literature including evidences from a wide range of human populations with different study designs and dietary measurements, and also evidence from animal and cell experiments (64). An advantage of DII is that it takes the whole diet into account, not just individual nutrients or foods (19). Previous studies have substantiated the utility of DII as a tool to characterize the inflammatory potential of diets and to predict the risk of multiple health conditions including colorectal cancer, cardiovascular diseases, and depression (65–68). Furthermore, DII was also used in epidemiologic studies to assess the potential association between diet and skeletal muscle aging.

All the included studies had observational design in nature, which were susceptible to confounding factors. Our findings were independent of certain confounding factors since most of the studies involved in meta-analysis adjusted their results for age, gender, and physical activity. However, residual confounding by some unmeasured factors and other unknown factors cannot be ruled out. For instance, the majority of included studies reported incomplete adjustment for some important confounders such as energy intake and comorbidity.

How diet associates with skeletal muscle aging can be partly explained by the systemic chronic inflammation that may lead to anabolic resistance and muscle stem cells (MuSCs) dysfunction (69–71). A systematic review and meta-analysis found that systemic inflammatory cytokines [including, CRP, IL-6, and tumor necrosis factor α (TNFα)] were negatively associated with muscle strength and muscle mass (15). Specifically, the dysregulated systemic chronic inflammation activates the ubiquitin-proteasome system by inhibiting the activity of insulin-like growth factor 1 (IGF-1) (72), leading to anabolic resistance and loss of muscle homeostasis in the aged people (69, 73). In chronic systemic inflammation, an increase in both M1 pro- and M2 anti-inflammatory macrophages was observed (74). The increased M1 macrophages account for higher levels of pro-inflammation cytokines (e.g., IL-1β, TNF-α, Interferon-γ). These cytokines will result in muscle dystrophies by impairing the regenerative function of resident MuSCs (70, 71). M2 macrophages can induce extracellular matrix accumulation and muscle fibrosis and impair the function of MuSCs, so as to affect skeletal muscle regeneration (75, 76). Given the information mentioned above, it is not surprising to find a positive association between pro-inflammatory diet (high DII) and skeletal muscle aging.

### 4.3. Strengths and limitations

In this study, we performed a systematic literature search across several bibliographic databases and included 24 observational studies in our meta-analysis. To the best of our knowledge, our work provided up-to-date finding on the associations between DII and skeletal muscle aging. More specifically, by taking into account muscle strength, muscle mass, and sarcopenia, we delivered an overview of evidence regarding how DII was related to skeletal muscle decline in adults. However, our work was also subjected to several limitations. Firstly, many of the included studies were cross-sectional designed, so causal conclusions could not be established based on our analysis results. Therefore, the findings require further validation by longitudinal or interventional studies. Secondly, the findings should be interpreted cautiously since evidence of publication bias was identified in the results of muscle strength, muscle mass, and sarcopenia. Nevertheless, the publication bias may be unreliable due to the small number of included studies in some outcomes (i.e., low muscle strength, muscle strength, and low muscle mass), and this may be changed with the increase of evidence in the future. Thirdly, several studies estimated muscle mass using BIA. Although BIA was validated as comparable to DXA (77), our meta-analysis may suffer from different equations that were used to estimate muscle mass. Finally, substantial heterogeneity was observed in certain groups. Our subgroup analyses suggested that region, age, and BMI were important sources of heterogeneity. However, residual heterogeneity was still observed. Previous studies implied that the number of dietary components used for DII calculation and the definition of sarcopenia might introduce significant inter-study heterogeneity, but the insufficiency of studies limited the power of such subgroup analyses (22, 39, 78). Therefore, more evidence with consistent methods for DII assessment and sarcopenia diagnosis is required to improve analysis and identify the sources of heterogeneity.

### 4.4. Clinical and research implications

Numbers of factors are responsible for malnutrition in older adults (79, 80), and malnutrition is a major risk factor for the age-related skeletal muscle decline (81). Sufficient nutrition plays a fundamental role in preserving skeletal muscle strength, mass, and function in older adults (82). Some evidence suggested that healthier diet patterns with adequate consumption of proteins, antioxidant nutrients, and long-chain polyunsaturated fatty acids exerted a positive effect on the prevention of skeletal muscle loss (82). However, the relationship between the dietary inflammatory potential and skeletal muscle was less clear.

In this study, a positive association between pro-inflammatory diet (high DII) and loss of skeletal muscle was observed. Based on this, a diet strategy with increased intake of anti-inflammatory dietary components (e.g., vegetables and fruits) and decreased intake of pro-inflammatory components (e.g., sugar-sweetened drinks and processed meat) is expected to be preventive for skeletal muscle health. Our finding suggested that the DII should be cautiously considered in formulating nutritional intervention recommendations for older adults from the aspect of skeletal muscle loss management. We also implied the utility of DII as a tool to predict the risk of skeletal muscle loss. Moreover, our results reinforced the public awareness of the pro-inflammatory property of diet and the need to avoid exposure to the risk of inflammation, and highlighted the rationale for DII control for the purpose of preventing skeletal muscle loss in older adults.

## 5. Conclusion

In summary, our meta-analysis suggested that higher dietary inflammatory potential was significantly associated with lower skeletal muscle strength, mass, and higher prevalence of sarcopenia. A larger number of longitudinal or interventional studies with consistent assessment and standardized methodology are needed to further explore the association between dietary inflammatory potential and skeletal muscle in the future.

## Data availability statement

The original contributions presented in this study are included in the article/Supplementary material, further inquiries can be directed to the corresponding author.

## Author contributions

NW and HX conceived the study. HW and HX were responsible for the design of the study and study selection and did the data extraction and risk of bias assessment. YL, WL, and ZW contributed to preparation and data analysis. NW contributed to the revision of the manuscript. All authors contributed to the article and approved the submitted version.
